# FAD: a comprehensive database merging information about food allergens

**DOI:** 10.1093/database/baag028

**Published:** 2026-05-27

**Authors:** Wafa Mokhtari, Souad Khemili-Talbi, Jean Kwasigroch, Dimitri Gilis

**Affiliations:** Computational Biology and Bioinformatics Lab, École polytechnique de Bruxelles, Université libre de Bruxelles, Avenue F.D Roosevelt 50, 1000 Brussels, Belgium; Biological Resources Valorization and Conservation Laboratory ‘VALCORE’, Department of Biology, M’Hamed Bougara University of Boumerdes, Avenue de l’independance, 35000 Boumerdes, Algeria; Laboratory of Bioinformatics, Applied Microbiology and Biomolecules (BMAB), Department of Nature and Life Sciences and Department of Agronomy, M’Hamed Bougara University of Boumerdes, Avenue de l’independance, 35000 Boumerdes, Algeria; Computational Biology and Bioinformatics Lab, École polytechnique de Bruxelles, Université libre de Bruxelles, Avenue F.D Roosevelt 50, 1000 Brussels, Belgium; Computational Biology and Bioinformatics Lab, École polytechnique de Bruxelles, Université libre de Bruxelles, Avenue F.D Roosevelt 50, 1000 Brussels, Belgium

## Abstract

Food allergies represent a significant and growing global challenge. However, researchers and risk assessors still lack a dedicated and comprehensive resource that centralizes food allergen information. As a result, obtaining the necessary structural, biochemical, and immunological data often requires time-consuming searches across multiple heterogeneous databases, hindering efficient analysis and slowing scientific progress. The Food Allergen Database (FAD) merges biochemical, structural, immunological, and functional properties of all known food allergens and isoallergens (1168 entries). It is an essential resource that facilitates the extraction of all information about food allergens.

## Introduction

Food allergies are a common and increasingly prevalent problem worldwide, affecting populations in both industrialized and developing countries [1–6]. The clinical spectrum of allergic reactions ranges from mild, self-limited manifestations to severe, potentially fatal anaphylactic shock, exerting a substantial impact on patients’ quality of life. Food-induced anaphylaxis represents one of the primary causes of hospital admission in the paediatric population [7]. Additionally, food allergies in children can potentially lead to severe consequences for their growth, physical health, and psychosocial well-being [8].

Given the importance of understanding the global burden of food allergies, a number of databases have been established to gather available information about allergens. Allergome is a comprehensive database that provides detailed annotations for each allergen based on the literature [9]. The COMPARE [10] and AllergenOnline [11] databases share substantial overlap in content, as both provide curated lists of allergenic proteins based on NCBI identifiers and offer tools for assessing potential cross-reactivity through sequence comparison. AllergenOnline includes protein sequences from allergen sources such as food, respiratory, contact, venom, and salivary allergens with documented IgE binding. In addition to these publicly available sequences, COMPARE also incorporates unpublished predicted protein sequences. The International Union of Immunological Societies (IUIS), under the auspices of the World Health Organization (WHO), manages the database responsible for standardizing allergen nomenclature, including food allergens, respiratory allergens, and others [12]. The Structural Database of Allergenic Proteins (SDAP 2.0) [13] includes a dedicated section that lists sequences and structures of food allergens classified by the Allergen Nomenclature Sub-Committee of the WHO and IUIS. Finally, the only database developed to provide information on allergenic foods and food allergens is InformAll [14]. It was created in 2006 but has not been updated since 2009. Despite the availability of several databases, none focuses exclusively on food allergens, and none offers an easy, integrated access to sequence, structure, and epitope data simultaneously. To gather all this information, we currently need to search in one of the allergen databases mentioned above, as well as in the Protein Data Bank [15] or the AlphaFold Structure Database [16] for structural information, the Immune Epitope Database (IEDB) for epitopes [17], UniProtKB [18] for complementary annotations, and InterPro [19] or Pfam [20] to obtain the family of an allergen. Given that millions of people worldwide are affected by food allergies, it is essential that scientists have access to a specialized database on food allergens, which gathers sequence, structure, and epitope information. This is the objective of FAD, the Food Allergen Database.

## Materials and methods

### Database build process

**Figure 1:**
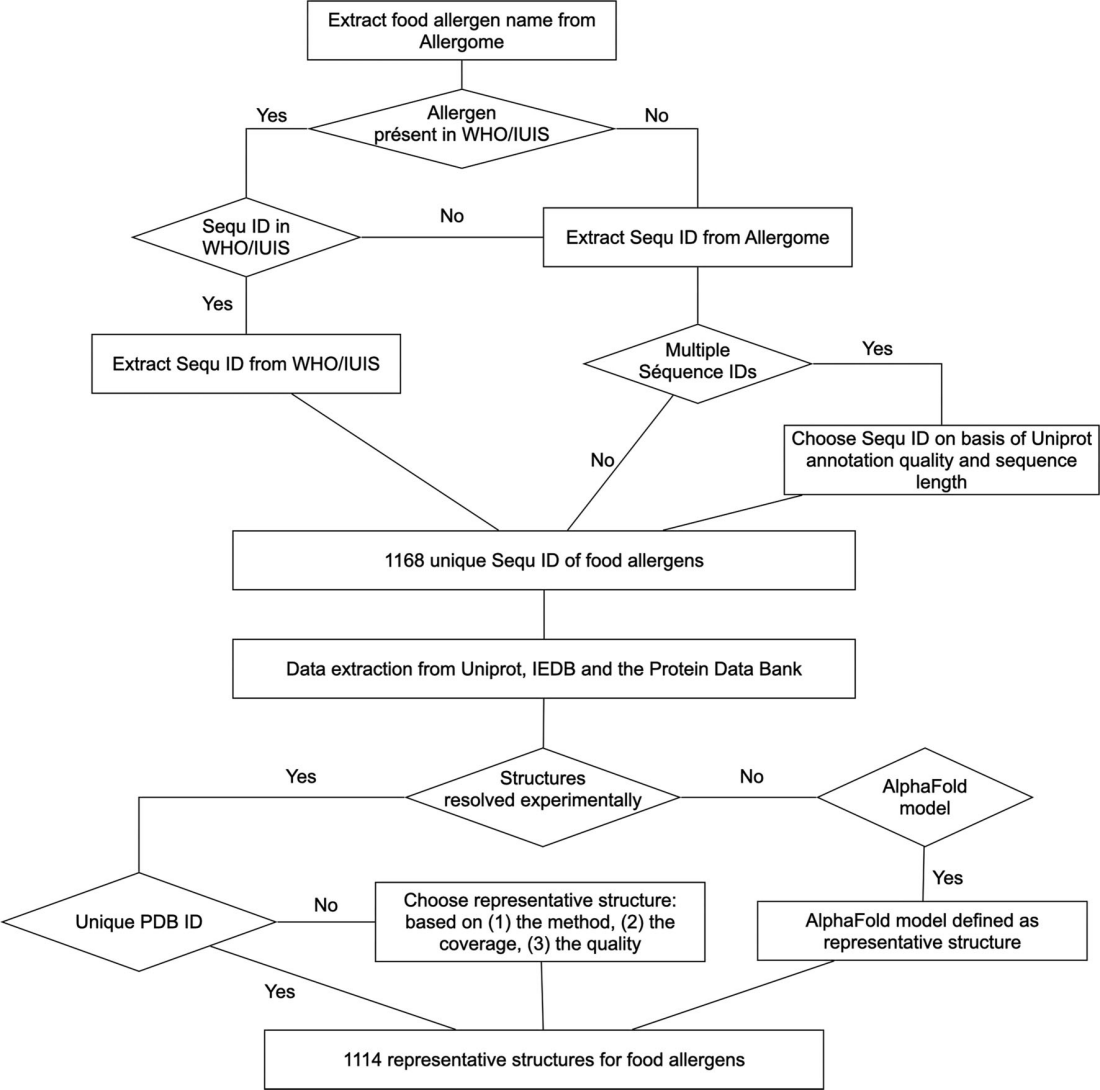
Flowchart of the pipeline followed to integrate food allergens into FAD.

The Food Allergen Database (FAD) is constructed by gathering data dispersed across different databases. Our primary data sources are the Allergome database [9] and the WHO/IUIS Allergen Nomenclature Database [12]. The WHO/IUIS database corresponds to the official allergen nomenclature and currently contains 1148 allergens, including 410 food allergens. Allergome, by contrast, lists 7542 allergens, of which 957 are food allergens, and includes additional entries that have been experimentally verified but are not present in the WHO/IUIS database. We selected these two databases as primary sources because they provide complementary and experimentally supported information: WHO/IUIS offers the authoritative nomenclature framework, while Allergome supplies a broader range of experimentally validated allergen records and additional descriptive data. In comparison, the COMPARE database includes 2881 allergens, but several are annotated as putative or predicted, and AllergenOnline contains 2372 allergens, including 838 food allergens, although the diversity of information associated with each allergen is more limited than in Allergome. Our objective was therefore to integrate into FAD a set of experimentally supported and biologically relevant data drawn from these complementary sources (WHO/IUIS and Allergome) to facilitate the study of food allergens (Fig. 1).

#### Selecting representative sequences

To compile a comprehensive and non-redundant dataset of food allergens, a hierarchical selection protocol was implemented. For allergens listed in the WHO/IUIS Allergen Nomenclature database, the corresponding UniProt or NCBI accession numbers were retrieved directly. For allergens not present in the WHO/IUIS database, the Allergome database was utilized as the secondary source. In cases where a single allergen in Allergome was linked to multiple identifiers, the entry associated with the UniProt record possessing the highest annotation quality was selected. If annotation qualities were identical, the entry corresponding to the longest protein sequence was chosen. This methodology resulted in a final dataset comprising 1168 unique food allergens and isoallergens, of which 576 were derived from the WHO/IUIS database.

#### Data extraction from UniProt and the Protein Data Bank

Once the UniProt code of the allergen is identified, we extract various information from UniProt and the Protein Data Bank (PDB). For each food allergen, UniProt will provide details including sequence length, annotation quality (reviewed and unreviewed), taxonomy identifier (tax ID, including scientific and common names of the organism) [21, 22], gene ontology annotations [23, 24], superfamily identifiers (SupFam) [25, 26], PDB codes, and AlphaFold codes. For each structure present in the PDB, we extract the method used to resolve the three-dimensional (3D) structure of the food allergen, the resolution and R-factor for structures resolved by X-ray crystallography, the chains corresponding to the allergen, and the boundaries of the sequence region to which the structure corresponds.

#### Selection of a representative structure

In the case where an allergen has multiple structures in UniProt and the PDB, we identify a representative structure based on the following decision tree. We first prioritize structures resolved by X-ray crystallography, then by nuclear magnetic resonance (NMR), and finally those modelled by AlphaFold. If multiple structures remain after this first criterion, we choose the structure that corresponds to the largest part of the allergen sequence. Next, the structure with the best resolution, and then the one with the best R-value (in the case of structures resolved by X-ray crystallography).

FAD contains 1114 food allergens and isoallergens with a representative structure. There are 54 food allergens that do not have a representative structure. These are allergens with short sequences (≤20 amino acids), or sequences with unknown residues (marked as X).

#### Extraction of data from the Immune Epitope Database (IEDB)

We extracted epitopes corresponding to food allergens from the Immune Epitope Database (IEDB) [17]. FAD contains 2456 epitopes derived from food allergens and isoallergens, annotated by both structural and immunological types. Each epitope entry includes its IEDB identifier, its structural type (linear or discontinuous/conformational), and whether it was identified through a B-cell or T-cell assay. Of these epitopes, 1956 are B-cell epitopes (79.6%) and 500 are T-cell epitopes (20.4%), enabling users to filter the dataset according to immune response specificity and to focus on IgE-relevant epitopes when investigating allergenic mechanisms. For linear epitopes, the start and end positions within the allergen sequence, as well as the epitope sequence itself, are provided. For discontinuous epitopes, the list of amino acids involved in the epitope is reported.

### The web interface

FAD offers a user-friendly web interface for searching, browsing, and visualizing food allergen data and structures. Users can easily access information by entering a UniProt ID (e.g. P24296), the allergen name (e.g. Tri a 14), or part of the allergen name (e.g. Tri a); in the latter case, a table listing all allergens matching the specified name fragment is provided. Users can also access a table containing all allergens included in FAD. This complete table can be filtered by the common name of the allergen source, enzyme family, superfamily, or the tissue from which the allergen originates.

For the visualization of the 2D representative structure and the mapping of epitopes of food allergens on the biological assembly, we utilized 3Dmol.js [27]. The 3D structure and the amino acid sequence are fully interactive, and selecting a residue in either view highlights it simultaneously. For AlphaFold structure, a Predicted Aligned Error (PAE) heatmap is also included and rendered via Plotly.js [28]. This allows users to critically evaluate the confidence in the relative orientation of structural domains and interactively link the PAE map back to the 3D structure, ensuring researchers have all the necessary quality metrics to assess and utilize the food allergen’s structural information for immunological studies.

The content of FAD can be downloaded in CSV format, and the fields to be included in the file can be customized.

#### Page displaying the complete database table

The ‘Entire Database’ page presents a table organized by allergen name, featuring eight fields: allergen name, common and scientific names, Uniprot and NCBI IDs, enzyme classification ID, SupFam ID, and allergenic tissues. Users can download this entire database and further refine their search using a filter based on four criteria (common name, allergenic tissue, enzyme classification, or superfamily) with an ‘or’ condition.

## Database statistics

To provide an overview of FAD through key figures, the database comprises 1168 unique food allergens and isoallergens. Of these, 576 are recognized by the WHO/IUIS Allergen Nomenclature database, while the remaining 592 are sourced exclusively from Allergome. Structural information is available for 1114 entries (95.4%): 140 allergens (12.0%) have at least one experimentally determined 3D structure—obtained by X-ray crystallography, NMR, cryo-EM, or neutron diffraction—whereas 974 (83.4%) are represented by an AlphaFold model. In total, 2456 epitopes are mapped across 87 allergens (7.4% of all entries), including 1956 B-cell epitopes (79.6%) and 500 T-cell epitopes (20.4%).

## Results and discussion

To illustrate one of the uses of FAD, we will take as an example an allergen from wheat, Tri a 14. The general information page is organized into sections: Allergen information, Sequence information, Gene Ontology, InterPro classification, and SupFam sequence superfamily. These sections provide detailed information, indicating, e.g., that Tri a 14 is an allergen whose nomenclature is recognized by WHO/IUIS, that it is a non-specific lipid transfer protein of 113 amino acids expressed in wheat. Its InterPro classification is IPR036312 ‘Bifunctional inhibitor/plant lipid transfer protein/seed storage helical domain superfamily’. The superfamily is identified as SSF47699 ‘Bifunctional inhibitor/lipid-transfer protein/seed storage 2S albumin’. Its UniProt code is P24296, with a link to the sequence; a link to the Allergome database is provided. The Gene Ontology annotations describe the functional roles of the allergen, such as lipid binding (GO:0008289) and lipid transport (GO:0006869). These elements provide a comprehensive overview of the molecular and functional characteristics of the allergen.

The ‘Structure’ tab in FAD is designed to offer an adaptive and interactive visualization of food allergen structural data, whether obtained experimentally or predicted computationally. For Tri a 14, the representative structure—defined according to the criteria described in the Methods—is the PDB entry 1BWO. This structure was solved by X-ray crystallography at 2.10Å resolution, with an R-value of 0.213. Additional structural data are also available for Tri a 14, including 1CZ2, 1GH1, as well as an AlphaFold model. The 3D visualization is powered by the 3Dmol.js module [27].

In cases where a food allergen lacks experimentally determined structural data (Bos d 5, for instance), the representative structure is the AlphaFold prediction. The pLDDT score is displayed as the central global quality metric [29]. This score also drives the core visualization: the predicted structure is coloured on a gradient ranging from blue (high confidence) to red (low confidence), providing an immediate sense of local prediction reliability.

**Figure 2:**
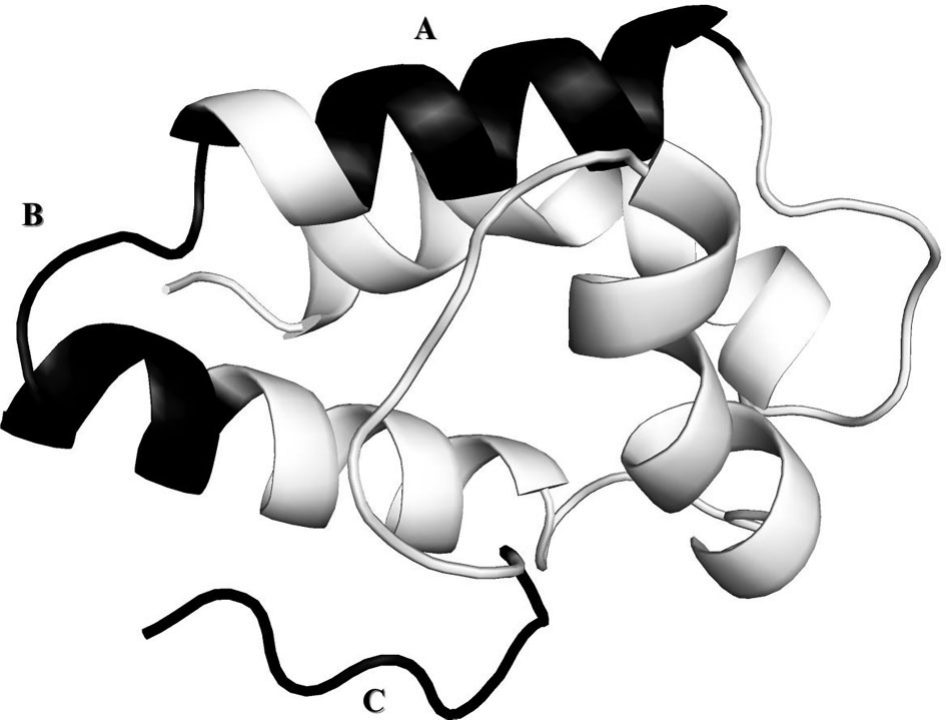
The image depicts the mapping of three Tri a 14 linear epitopes on the representative structure 1BWO, visualized using PyMOL [31]. The epitopes are highlighted in black, where ‘A’ corresponds to the epitope sequence GQCCDGVKNL and is located on a helical region, ‘B’ corresponds to the epitope sequence QARSQSDRQS, also located on a helical region, and ‘C’ corresponds to the epitope sequence ISLNIDCSRV, situated on the C-terminal part of the representative structure 1BWO.

Epitope mapping allows the identification of specific regions within the allergen that are recognized by the immune system, thus facilitating a deeper understanding of allergenicity. The ‘Epitope’ tab informs us that three epitopes have been identified for Tri a 14: GQCCDGVKNL (sequence limits: 48–57; limits in the structure 1BWO: 25–34), QARSQSDRQS (sequence limits: 60–69; limits in the structure 1BWO: 37–46), and ISLNIDCSRV (sequence limits: 104–113; limits in the structure 1BWO: 81–90). The integration of structural data with epitope information is a cornerstone of allergen immunology research. Having both structural information and epitope data is crucial, as it allows researchers to study the spatial arrangement of epitopes within the allergen and assess their accessibility and immunogenic potential. This knowledge is fundamental for the development of precision diagnostics and therapeutic strategies, including epitope-based profiling to predict tolerated allergen doses [30] and allergen-specific immunotherapies. While the generation of hypoallergenic variants remains a potential avenue in specific, well-defined contexts, it must be approached with caution given the current limitations in fully eliminating all major allergenic proteins from complex food sources. Figure 2 shows the mapping of Tri a 14 epitopes on its representative structure 1BWO, using the link provided on FAD that allows matching the sequence numbering in UniProt to that of the PDB. This enables the exploration of the immunological properties of allergens. As mentioned above, the superfamily of Tri a 14 is SSF47699. The FAD interface also allows users to search for all allergens that belong to the same superfamily as Tri a 14. To do this, the user can return to the homepage, request access to the entire database with the ‘Full’ code, and then filter the table with the superfamily code. The table presenting all allergens belonging to the SSF47699 superfamily, ‘Bifunctional inhibitor/lipid-transfer protein/seed storage 2S albumin’ is therefore provided. The obtained results can be downloaded from the website. They show that this superfamily of lipid transporters contains a large number of allergens from various organisms.

The FAD also offers flexible download options, including full access to the entire database. Users can download the complete dataset by clicking on ‘Advanced Download of the entire database’ from the page displaying the entire database table. For customized downloads, specific fields can be selected from a table, generating the desired dataset.

Additionally, users can access supplementary data, such as epitopes referenced in IEDB, representative 3D structures, or experimental and modelled PDB files of allergens, through the ‘Additional Downloads’ option.

Although FAD was designed to provide a broad and integrated view of food allergens and their epitopes, several aspects of the resource naturally reflect the current landscape of available data and thus represent opportunities for future development.

A first area for expansion concerns cross-species epitope exploration. At present, epitopes are mapped to individual allergen entries, but explicit links between homologous proteins from different species are not yet incorporated. Introducing such cross-reactivity relationships would greatly benefit studies on panallergens and shared sensitization patterns, and represents a promising direction for future releases.

In addition, FAD is conceived as a curated, integrative database rather than a predictive platform. For this reason, it does not currently include built-in allergenicity prediction tools. However, the modular structure of the resource makes it well suited for the incorporation of computational models, and the integration of predictive functionality is planned as a natural next step.

It is also worth noting that FAD relies entirely on high–quality upstream sources—WHO/IUIS, Allergome, UniProt, PDB, AlphaFold, and IEDB—rather than performing de novo experimental curation. This approach ensures consistency with established community standards, while inherently reflecting any limitations or biases present in these source databases.

Finally, epitope coverage varies across allergen entries, with some well-studied allergens benefiting from rich experimental characterization while others remain sparsely annotated. This variability mostly mirrors the current state of the literature and highlights areas where additional experimental work could further enrich the field.

## Conclusion

It is important to place the development of our FAD within the broader context of existing allergen databases. Currently, several allergen databases are available, each offering different types of data and organizing them in diverse ways. Notable examples include the SDAP 2.0 Structural Database of Allergenic Proteins [13], Allergen Online [11], and InformAll [14], the latter, unfortunately, no longer being updated. The study of food allergens has long been hampered by the fragmentation and dispersed nature of essential data across numerous, non-specialized databases. To address this fundamental challenge, the FAD has been developed as a centralized and dedicated resource designed to unify key data types previously held in informational silos.

The construction of FAD is founded on a systematic and rigorous curation pipeline that ensures the inclusion of a high-quality, non-redundant dataset. This methodology relies on a hierarchical sourcing protocol, prioritizing the WHO/IUIS Allergen Nomenclature database and complementing it with data from Allergome. FAD stands out through its dedicated focus on food allergens and its comprehensive integration of biochemical, structural, and immunological data within a single platform. It provides a systematically selected representative 3D structure for food allergens, drawing from both experimentally resolved structures and high-confidence AlphaFold models. In addition, FAD compiles curated experimental epitopes from the Immune Epitope Database (IEDB) [17].

Through its interactive web interface, advanced filtering features, and flexible download options, FAD streamlines what is typically a complex and time-consuming process of gathering and organizing allergen-related information. Ultimately, this unified platform facilitates a more holistic understanding of food allergen properties, providing the scientific community with a robust and reliable resource to enhance reproducible research.

FAD is positioned to foster new insights into the structural and immunological determinants of food allergies, thereby serving as a foundational tool to accelerate research into the mechanisms of allergenicity and support the development of novel diagnostics and immunotherapies. The database is publicly accessible and will be maintained with regular updates to incorporate new findings in the field. Future updates are also expected to integrate a predictive tool for assessing the allergenicity of proteins used in the food industry, further extending the analytical capabilities of the platform.

## Future development

FAD development will continue with regular updates incorporating newly curated food-related allergen data. In addition, FAD will integrate a computational prediction tool designed to assess the allergenic potential of food-related proteins.

## Data Availability

The web server underlying this article is available freely online at http://babylone.ulb.ac.be/faddatabase/
